# Experiential learning through virtual reality by-proxy

**DOI:** 10.1007/s10055-025-01106-3

**Published:** 2025-02-08

**Authors:** Nicola Veitch, Claire Donald, Andrew Judge, Christopher Carman, Pamela Scott, Sonya Taylor, Leah Marks, Avril Edmond, Nathan Kirkwood, Neil McDonnell, Fiona Macpherson

**Affiliations:** 1 School of Infection and Immunity, College of Medical, Veterinary and Life Sciences, University of Glasgow, Glasgow, Scotland; 2 School of Social and Political Sciences, College of Social Sciences, University of Glasgow, Glasgow, Scotland; 3 School of Molecular Biosciences, College of Medical, Veterinary and Life Sciences, University of Glasgow, Glasgow, Scotland; 4 School of Medicine, Dentistry and Nursing, College of Medical, Veterinary and Life Sciences, University of Glasgow, Glasgow, Scotland; 5 Glasgow, Scotland; 6 School of Humanities, College of Arts, University of Glasgow, Glasgow, Scotland; 7 Centre for the Study of Perceptual Experience, Philosophy, School of Humanities, College of Arts, University of Glasgow, Glasgow, Scotland

**Keywords:** Virtual reality, Practical skills, qPCR, Student experience, Remote learning, Microbiology, Educational research

## Abstract

Virtual reality (VR) is increasingly being used as a teaching and learning tool, however scaling this technology is difficult due to technological and cost considerations. An alternative approach that helps to address these problems is VR-by-proxy, where teaching takes place within a VR environment that is controlled by one lecturer and broadcast to students online. This allows the content to be accessed without specialist equipment while still offering an immersive and interactive experience. Taking advantage of the enforced move to online learning during the COVID-19 pandemic, this study evaluates the implementation of a novel VR-by-proxy disease diagnostic laboratory VR simulation within an undergraduate life sciences course in a higher education setting. Student participants were randomly allocated into two groups: the test group, who took part in a VR-by-proxy lesson; and a control group, who worked with interactive online lab manual material. We assessed improvement in learning and enjoyment through questionnaires before and after these tasks and collected qualitative data on student attitudes towards VR through focus groups. Our results indicate that although there is no observable difference in learning outcomes between the two groups, students in the test group reported an improved learning experience, confidence and enjoyment of learning. In our focus groups, confidence was understood in two ways by participants: firstly, as ‘understanding’ of the various steps involved in conducting a quantitative polymerase chain reaction experiment and secondly as a more general ‘familiarity’ with the laboratory setting. This study adds to the growing body of research into the effectiveness of VR for learning and teaching, highlighting that VR-by-proxy may provide many of the same benefits.

**Supplementary Information:**

The online version contains supplementary material available at 10.1007/s10055-025-01106-3.

## Introduction

Virtual reality (VR) has been adopted in various education-based settings and has followed the increased integration of digital technology within the field of education (Zawacki-Richter and Latchem 2018; Ray and Srivastava 2020). The coronavirus disease 2019 (COVID-19) pandemic led to a rapid implementation of online remote learning opportunities within Higher Education (HE) as educators worldwide quickly transformed their teaching, identifying what digital resources were available and adapting them accordingly (Tabatabai 2020). Innovative interventions were required to engage students in distance learning, so the use of novel technologies, such as VR in teaching, were developed (Toquero 2020).

Traditionally, VR has been delivered through two main methods, immersive VR (I-VR) using a headset, earphones and controller, and desktop VR (D-VR), viewed on a computer screen and controlled by a keyboard and mouse (Hamilton et al. 2021). VR-by-proxy is an approach where an individual controls the I-VR headset and broadcasts the simulation online via communication software. This method of teaching, and indeed teaching using VR more generally, is an exciting opportunity which has emerged during a time when innovative teaching approaches not only benefit learning (Pregowska et al. 2023), but also provided a sense of shared experience to increase a student’s sense of belonging and fulfilment (Osterman 2000) during the switch to remote learning.

It is well established that computer-based games and simulations are valuable training tools for the enhancement of a range of skills (Hays and Singer 1989; Faria and Wellington 2004; Al-Elq 2010; Perry et al. 2015; Makransky et al. 2020). The integration of VR technology into education follows the more general adoption of such technology within training in industry, science and engineering. This shift is no doubt partly explained by recent advances in the VR technology itself (Jensen and Konradsen 2018; Radianti et al. 2020), as well as the consequent drop in prices for VR hardware and software (Hodgson et al. 2015).

The benefits of integrating VR technology into an educational setting are easy to imagine. That is, through engaging with the virtual form of reality, this technology allows educators to cross many important pedagogical boundaries. For example, students can visit locations that would otherwise be unfeasible to reach, engage in situations that would be too dangerous for them to encounter (Wyk and Villiers 2009; Markowitz et al. 2018), make mistakes that would be too costly (Burns and Köste 2016), and engage with impossible situations that they could not confront in the real world (Ott and Freina 2015; Jones 2018). Moreover, there is a vital gap that students have to cross between the theoretical knowledge that they acquire and the relation of that knowledge to real-world situations, such as practical experimentation (Klingenberg et al. 2020). This is a boundary that VR appears likely to help students cross, as the constructivist theory of experiential learning, essentially ‘learning by doing’, would predict with multiple studies supporting the idea that immersive technology increases understanding of complex topics (Salzman et al. 1999; Jensen and Konradsen 2018; Makransky et al. 2019; Zhao et al. 2020; Coban et al. 2022; Ryan et al. 2022).

Research into the effectiveness of VR has demonstrated multiple benefits that can be gained from its implementation into educational contexts; including increased motivation compared to less immersive media (Makransky and Lilleholt 2018; Klingenberg et al. 2020) as well as increased engagement and skills acquisition (Loup et al. 2016; Conrad et al. 2024).

In considering the effectiveness of I-VR in improving the student learning experience, barriers to that learning also need to be considered to make the technology inclusive. One reported barrier is cybersickness (McCauley and Sharkey 1992; Weech et al. 2019), where users feel nausea and motion sickness when using the technology, with several factors considered to contribute, including: quality of hardware, time delay and flickering displays (Chang et al. 2020). Investigations into how VR-by-proxy affects nausea ought to inform the future use of the technology in education settings, in order to create an inclusive curriculum.

While research into the effectiveness of VR for learning and teaching has been emerging recently in education and psychology literature, this area of research is still in its relative infancy. As the quality and affordability of available VR systems improves, its attractiveness as a teaching tool is expected to increase. This, in combination with the post-pandemic drive observed in HE to restructure approaches to teaching, illustrates a need for a better understanding of how VR, and other similar technologies, can be adopted to have the greatest benefit on student learning. Hence, examining the effectiveness of the innovative VR-by-proxy method for learning is an important and timely topic that should help inform future practice and further the efficiency of a vital and evolving field of research.

### Research questions

Based on previous literature, this study focussed on five key research questions, utilising questionnaires and focus groups to evaluate student groups:

- Does exposure to a simulated VR-by-proxy lab enhance student learning in relation to theoretical knowledge?
- Does VR-by-proxy improve students’ self-reported learning experience?
- Is confidence in student learning improved after exposure to a VR-by-proxy lab?
- Does a VR-by-proxy lab experience enhance student enjoyment of learning?
- Do students that report nausea during VR-by-proxy experiences have a diminished learning experience?

Drawing on the extant literature on VR available, five hypotheses were developed linked to our research questions and overall aim of examining the effectiveness of VR-by-proxy:

- H1: Exposure to the simulated VR laboratory will have no effect on student achievement of learning outcomes
- H2: Exposure to the simulated VR laboratory will improve students’ self-reported learning experience
- H3: Exposure to the simulated VR laboratory will improve students’ self-reported confidence in their learning
- H4: Exposure to the simulated VR laboratory will improve students’ self-reported enjoyment of learning
- H5: Students reporting a feeling of nausea will have a diminished learning experience

## Material and methods

The main aim of this work was to evaluate the implementation of a bespoke VR-by-proxy simulation with pre-Honours Life Science students, delivered within an HE setting during the COVID-19 pandemic. I-VR was not possible during the COVID-19 pandemic due to lockdown restrictions, where students and staff were not able to be on campus. In addition, obtaining and distributing multiple VR headsets to each student was cost prohibitive. VR-by-proxy allowed students to have a shared experience, whereby students were in an online group environment and could discuss questions with staff and peers. As part of the VR-by-proxy experience, staff utilised I-VR technology to broadcast an immersive lesson via the communication software Zoom to students.

### Materials

The materials involved in this study consisted of a virtual laboratory simulation (the Disease Diagnostic Laboratory) demonstrating appropriate quantitative polymerase chain reaction (qPCR) practice delivered by-proxy, the Moodle laboratory manual, three participant questionnaires (S1 File) and focus groups (S2 File).

#### Virtual reality simulation

This study used the ‘Disease Diagnostic Laboratory’ VR app which was designed and developed by the authors in collaboration with immersive VR and augmented reality (AR) company, Edify. A voice-over walk through of the simulation is available (S3 File). The simulation covered several technical concepts, key skills and was linked to specific intended learning outcomes (ILOs) (Table 1).

**Table 1:** Lists of key concepts, skills and intended learning outcomes covered by the disease diagnostic laboratory VR app

Technical concepts	Key skills	Intended learning outcomes
Aseptic technique	Working independently within a laboratory	Compare and contrast PCR and qPCR
Using pipettes	Familiarity with laboratory environment and equipment	Understand the process of qPCR and its diagnostic use
Using a microbiological safety cabinet		Learn how to analyse qPCR results using R studio
Preparation of a master mix		

The simulation was delivered to participants using VR-by-proxy via Zoom and included input from two members of staff; one to virtually enter the VR laboratory wearing the VR headset and remotely guide students through the simulation, and the second to monitor the Zoom chat function which students used to ask questions and receive answers in real time throughout the experience. The simulation took approximately 20 min to complete.

**Fig. 1:**
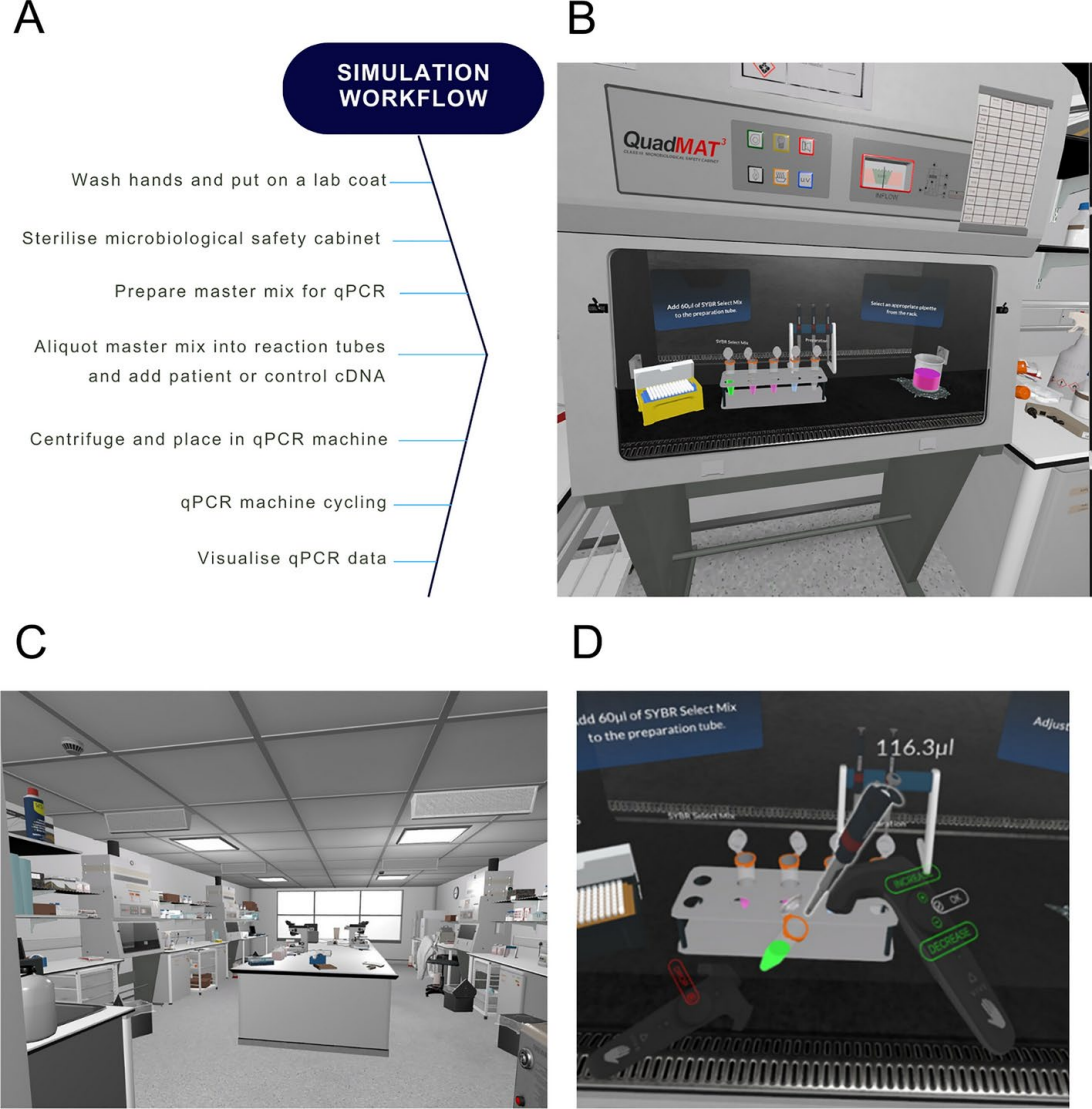
The disease diagnostics laboratory virtual environment. The experimental workflow to set up a qPCR experiment (**A**) and user views of the environment and available equipment (**B**–**D**)

The Disease Diagnostic Laboratory VR app allows users to experience the processes involved in diagnosing a viral disease using qPCR within a virology laboratory environment. The aim of the experience is to quantify the concentration of viral nucleic acid present in the samples using qPCR and compare those results to positive and negative control samples to diagnose each patient. The simulation covered each step of the experimental workflow (Fig. 1A), from appropriate hygiene practices upon entering the laboratory and putting on a lab coat, to placing the prepared reactions into the qPCR machine and generating the data output (Fig. 1B–D). Students were unable to control the speed of pace of the simulation as this was dictated by the member of staff in the VR environment.

On completion of the simulation, students were able to view their results within the VR environment. This information was also included within the online Moodle lab book for analysis outside of the simulation and students were provided with staff support to enable them to analyse the data correctly using R-studio. At the end of the VR-by-proxy session, students were encouraged to reflect on their experience as a group, ask questions and then discuss this with staff.

#### Moodle laboratory manual

**Fig. 2:**
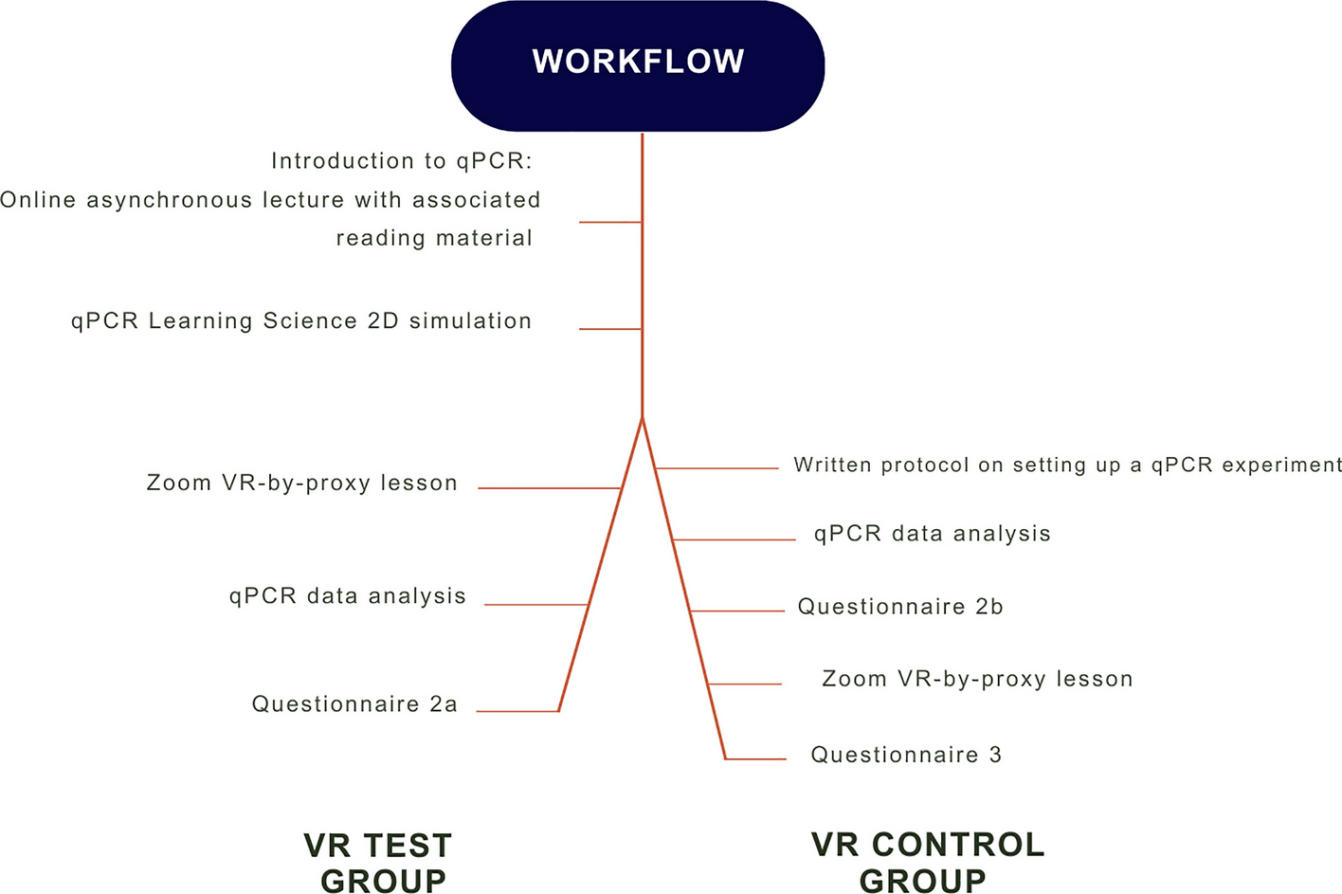
The online moodle lab manual workflow for both the VR-test group and the control group. Differences in tasks completed by the two groups are indicated

In the absence of the ability to be on campus during the 2020/2021 academic session due to the COVID-19 pandemic, online Moodle book lab manuals were created. Two versions of the lab manual were required linking to the two different VR-Test and Control groups (Fig. 2). These online lab manuals contained various reading materials, including text and diagrams, 2D interactive simulations, quizzes and explanatory videos. These also contained links to the VR-by-proxy Zoom lesson and questionnaires. Both lab books started by explaining the key learning outcomes and qPCR theory and subsequently allowed students to interact with a 2D ‘Learning Science’ qPCR simulation (S3 File). Following this, the VR-Test group linked to the VR-by-proxy lesson, while the VR-Control group read a written protocol on setting up a qPCR, which aligned to the ILOs of the VR simulation (Table 1), which the VR-Test group did not receive. The VR-Control group then worked through the data analysis followed by completing questionnaire 2b. Once this was accomplished they experienced the VR-by-proxy simulation. Both the test and control groups then completed their final questionnaire (2a and 3, respectively).

### Methods

#### Participants and design

**Fig. 3:**
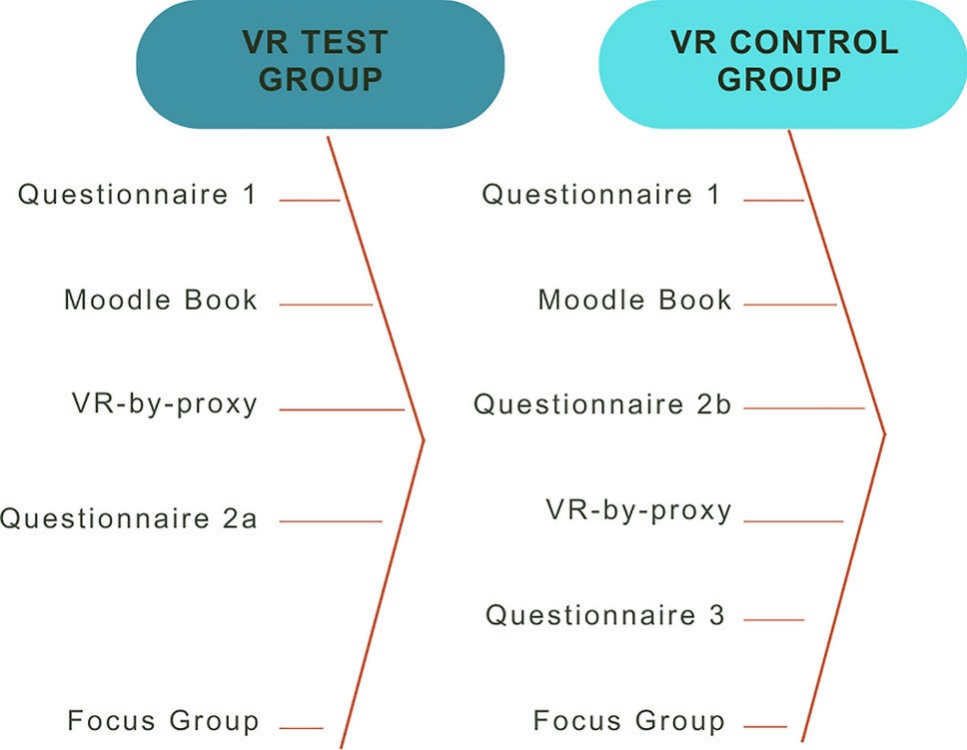
Schematic illustration of the study workflow for both groups

The study consisted of 76 participants who were enrolled as Level 2 and 3 undergraduate students on 4-year Life Science degrees at the University of Glasgow in 2020/2021. Participants were randomly allocated into two study groups which were both provided with an online laboratory manual within a virtual learning environment (Moodle) to work through at their own pace within a specified timeframe of 3 h. One group (VR-Test group; N = 39) observed a VR-by-proxy simulation demonstrating the specified laboratory technique as part of the lesson, while the second group (VR-Control group; N = 37) completed the online lab manual without the VR simulation (Fig. 3). The VR-Control group received a traditional online written protocol embedded into Moodle, which the VR-Test group did not receive, in place of the VR-by-proxy. Participants completed questionnaires before and after these tasks. Following this, the Control group participated in the VR simulation to ensure equality of access to learning tools and completed a final questionnaire to describe their experiences. In advance of the study, each participant was provided with relevant information and completed an online consent form permitting the use of their data within the study for both the questionnaires and the focus groups. Students were incentivised to join the study with the promise of the chance of receiving one of three £200 (GBP) Amazon gift vouchers, randomly allocated to participants who completed the questionnaires and participated in the focus group.

All students were required to fill in questionnaire 1 in advance of the Disease Diagnostics Laboratory VR lesson. Students in the VR-Test group completed the tasks in the Moodle book and then took part in the VR-by-proxy lesson delivered via Zoom. These students then completed questionnaire 2a and went on to take part in a focus group. Students from the VR-Control group first completed the tasks in the Moodle book and then questionnaire 2b. This was followed by the VR-by-proxy lesson, the completion of questionnaire 3 and the focus group.

Due to the COVID-19 restrictions, none of the participants obtained substantial lab experience prior to this study and would not have been in a teaching laboratory for the 12 months leading up to the study. Therefore, the majority of participants would have had no prior practical laboratory experience of the techniques covered in this study, although the theory had been discussed previously in lectures. This information was not gathered formally as part of the study but has been assumed from knowledge of the curriculum.

#### Questionnaire development

Questionnaires were developed by the research team to capture the key points of the 5 research questions and used a variety of question formats depending on the information type being gathered. Factual information (e.g., “What is the function of SYBR green in a qPCR?”, questionnaire 1) was largely gathered using multiple choice and true/false question formats. Following recommendations in Krosnick and Fabrigar 1997; Krosnick 1999; and Saris and Gallhofer 2007, evaluative assessments (e.g., “How confident would you feel working aseptically using a lamina flow hood?”, questionnaire 1) were measured using 7-point Likert-type bipolar items, including middle point response categories (Krosnick and Presser 2010, but see Johns 2006), with item-specific rating scales (Saris et al. 2010). We also included a limited number of open-ended format questions (S1 File). The questionnaires were fielded to participants using Google Forms at key points in the study as reflected in Fig. 3.

#### Pre-study questionnaire

Four questions linking to demographic information were asked to establish the participants’ gender, degree group and social class. Prior experience of using VR and knowledge of qPCR theory were ascertained to gain an understanding of the students’ previous experience of immersion and what they were aware of at the start of the study. Ten knowledge-based questions were posed: 4 questions to ascertain student confidence levels to perform qPCR experiments and 6 direct questions on qPCR theory.

#### Post-study questionnaires

All 10 direct knowledge-based questions were asked in both questionnaires 2a and 2b to determine what the students in the VR-Test group had learned during the VR lesson, supplemented by the Moodle lab book, and what those in the VR-Control group had learned from the online Moodle lab book, supplemented only by the written protocol. Specific questions relating to the two different learning scenarios were asked to assess student engagement, focussing on understanding, presence and involvement. Three questions probed student enjoyment of the scenarios relating to how likely students were to recommend the learning method and overall experience evaluations. Students were asked to rate how nauseous, if at all, they felt to determine if the VR lesson caused any learning barriers. The final questionnaire for the VR-Control group required students to consider which learning style they preferred and why. Five questions were asked around learning, immersion and nausea.

#### Focus group

Students in both groups who had completed all questionnaires issued to them were invited to participate in a focus group that took place following completion of the Disease Diagnostics Laboratory VR lesson. Focus groups were conducted to examine patterns of social meaning that participants attach to their experience of taking part in the VR-by-proxy laboratory. They are used to aid the interpretation of the quantitative results in the subsequent discussion section of this paper rather than as a means of testing hypotheses.

There were 6 focus groups in total, with 5–8 students randomly assigned to one of three focus groups for each of the two learning scenarios. One staff member was assigned to each focus group (one VR-Test and one VR-Control group) to facilitate discussion around a series of prompting questions on: (1) prior experience using VR; (2) support for learning key qPCR concepts using VR and the online Moodle lab manual; (3) confidence in using PCR in a real lab environment; and (4) enjoyability. All focus groups took place via Zoom and were recorded. Audio recordings and the text chat were transcribed, with comments raised by participants through both methods treated as equivalent. The resulting transcripts were fully anonymised and standardised durations calculated after the removal of non-substantive sections of the transcripts (Table 2).

**Table 2:** Focus group information

Group	Treatment or control	Participants	Facilitator	Standardised duration
1A	VR-Test	6	A	31:26
1B	VR-Test	5	B	26:18
1C	VR-Test	8	C	34:33
2A	VR-Control	6	A	31:35
2B	VR-Control	8	B	36:01
2C	VR-Control	7	C	37:07

## Results

### Questionnaire responses

#### Differences in learning outcomes

Our first hypothesis was that exposure to the simulated VR-by-proxy laboratory would have no effect on student learning in relation to theoretical knowledge. To test this, students were asked to respond to a series of fact-based, knowledge assessment questions in the post-test questionnaire. These questions presented students with multiple choice, true/false or ordering tasks. Responses were coded according to whether a student responded correctly (1) or incorrectly (0) to each item. We start our analysis by examining the bivariate relationships between the test groups (VR-Test or Control) to which students were assigned and each of the individual learning outcome measures. As both the test group and the learning outcome measures are dichotomous, non-parametric Pearson’s Chi-squared (χ^2^) tests were used to assess the underlying relationships between test group and learning outcomes variables. The analysis presented in Table 3 reveals no relationship between the group to which students were assigned and the learning outcome measures.^1^

**Table 3:** Learning outcome measures by test group

	VR-Test group		VR-Control group		
	Mean (SD)	Range	Mean (SD)	Range	χ^2^ (*p*)
Pipette	0.34 (0.48)	0, 1	0.24 (0.43)	0, 1	1.02 (*0.31*)
Sybr	0.34 (0.48)	0, 1	0.24 (0.43)	0, 1	1.02 (*0.31*)
Master mix	0.84 (0.37)	0, 1	0.76 (0.43)	0, 1	0.75 (*0.39*)
Contamination	0.84 (0.37)	0, 1	0.95 (0.23)	0, 1	2.24 (*0.14*)
Advantages	0.16 (0.36)	0, 1	0.13 (0.34)	0, 1	0.11 (*0.74*)
Cycle threshold	0.68 (0.47)	0, 1	0.74 (0.45)	0, 1	0.26 (*0.61*)

	VR-Test group		VR-Control group		Mean Dif (t)
	Mean (SD)	Range	Mean (SD)	Range	
Learning outcomes index	3.21 (1.3)	0–6	3.05 (1.2)	0–6	0.16 (0.56)

All *p* values are >0.05 and therefore not significant

**Fig. 4:**
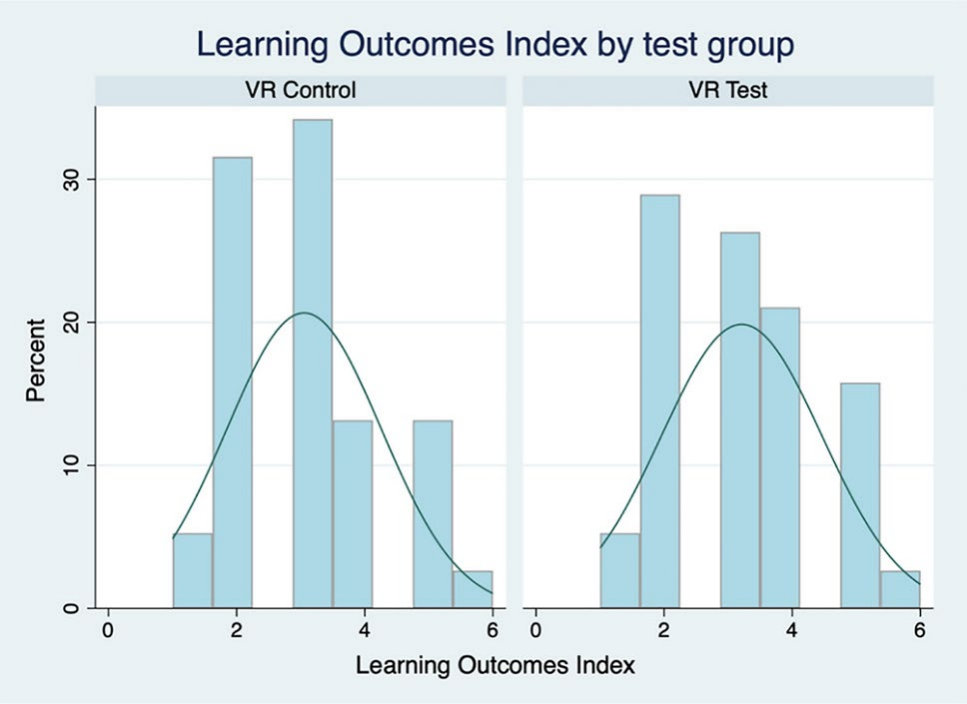
Distribution of learning outcomes index by test group

Additionally, we created an additive learning outcomes index (an additive index is the sum of the component items into a single measure) of the six learning outcome items (Range 1–6; Mean = 3.13; SD = 1.23). Combining the six component items into a single measure, we are able to visualise the overall cumulative differences in learning outcomes between the VR-Test group and the Control group, which we present in Fig. 4. A difference of means test further confirmed that there is no observable difference in learning outcome between the two groups (Mean Diff = 0.16; t = 0.56).

#### Differences in learning experience

Our second hypothesis was that exposure to the simulated VR-by-proxy laboratory would improve students’ self-reported learning experience. Table 4 presents the summary statistics for the six questionnaire items designed to assess student learning experience, each measured on a 7-point Likert scale. Students in the VR-Test group were asked to respond based on their experience with the simulated VR experience while students in the VR-Control group were asked to assess their experience with the online Moodle lab manual. Difference of means tests across the six individual items demonstrate that students in the VR-Test group consistently rated their learning experience more favourably (i.e., gave consistently higher mean ratings) than students in the VR-Control group did. All differences are significant at a minimum of the *p* < 0.05 level.

**Table 4:** Learning experience measures by test group

	VR-Test group		VR-Control group		
	Mean (SD)	Range	Mean (SD)	Range	Mean Dif (t)
Understanding	6.24 (.68)	5–7	5.95 (.61)	5–7	0.29* (1.96)
How present did you feel	5.74 (1.11)	3–7	4.92 (1.42)	2–7	0.82** (2.79)
Feel like in lab	5.13 (1.28)	1–7	2.79 (1.68)	1–6	2.34** (6.84)
Feel demonstrated in lab	5.74 (1.06)	3–7	3.13 (1.76)	1–7	2.61** (7.83)
Improved understanding	6.45 (.72)	4–7	5.63 (.85)	3–7	0.82** (4.50)
Recommend method	6.34 (1.10)	1–7	4.66 (1.58)	1–7	1.68** (5.39)
Experience index	29.63 (4.97)	12–36	21.08 (6.34)	7–34	8.55** (6.57)

*p <.05 **p <.01

**Fig. 5:**
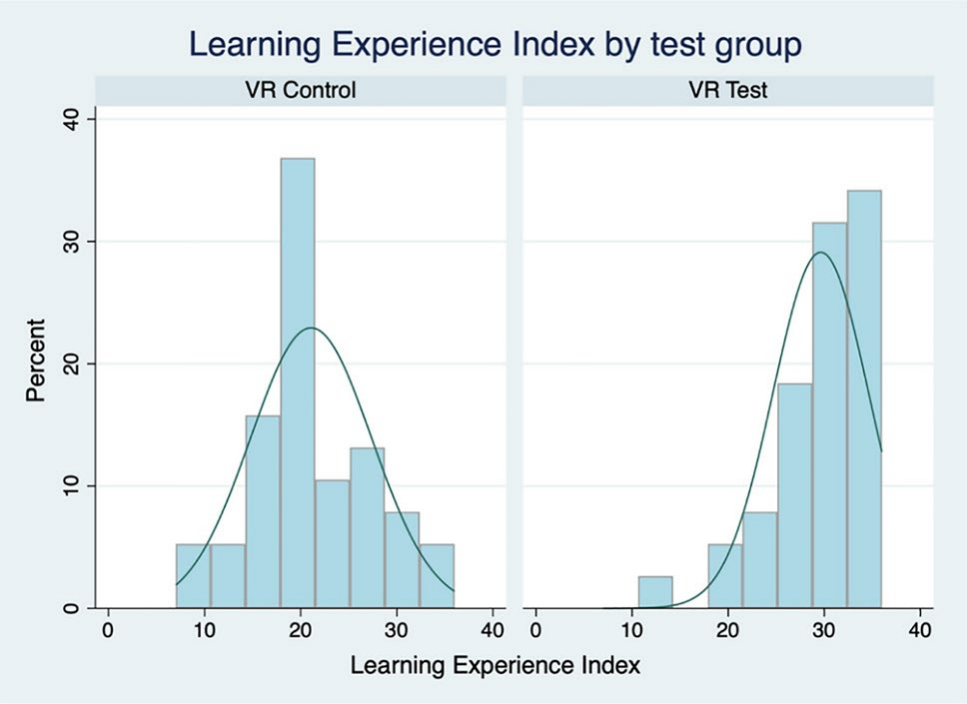
Distribution of the learning experience index by group

Table 4 also provides details of the additive Experience Index created using the six learning experience measures (Cronbach’s alpha = 0.89), with a theoretical range of 0–36. (Cronbach’s alpha is a reliability coefficient, often referred to as an indicator of internal consistency, that is conceptualised as the average inter-item covariance of the component measures). Figure 5 displays the distribution of the learning experience index by test group. The mean difference of 8.55 between the two groups is significant, well below *p* < 0.01. As hypothesised, there is a clear difference between the VR-Test and VR-Control groups, with those students exposed to the simulated VR laboratory reporting an improved learning experience.

#### Differences in confidence

Our third hypothesis was that exposure to the simulated VR-by-proxy laboratory would improve students’ confidence in their learning. Student confidence was assessed with three items, each measured on a 7-point Likert scale. Table 5 presents the summary statistics for these items and differences in means between the VR-Test and VR-Control groups. Two of the items, those assessing student confidence in performing a qPCR experiment independently and working aseptically using a lamina flow hood, are significantly different across the two groups. A third item, that which generally assesses confidence in understanding qPCR, is significantly different across the groups in a directional (one-tailed) test but falls short for a non-directional test.

**Table 5:** Learning confidence measures by test groups

	VR-test group		VR-Control group		
	Mean (SD)	Range	Mean (SD)	Range	Mean Dif (t)
Understanding	5.87 (0.11)	5–7	5.61 (0.12)	4–7	0.26 (1.66)
Perform qPCR independently	5.55 (0.14)	3–7	4.53 (1.33)	2–7	1.03** (3.99)
Working aseptically	5.71 (0.13)	4–7	4.32 (0.28)	1–7	1.39** (4.48)
Confidence Index	15.13 (0.34)	11–19	12.45 (0.54)	6–19	2.68** (4.39)

*p < .05 **p < *.01*

**Fig. 6:**
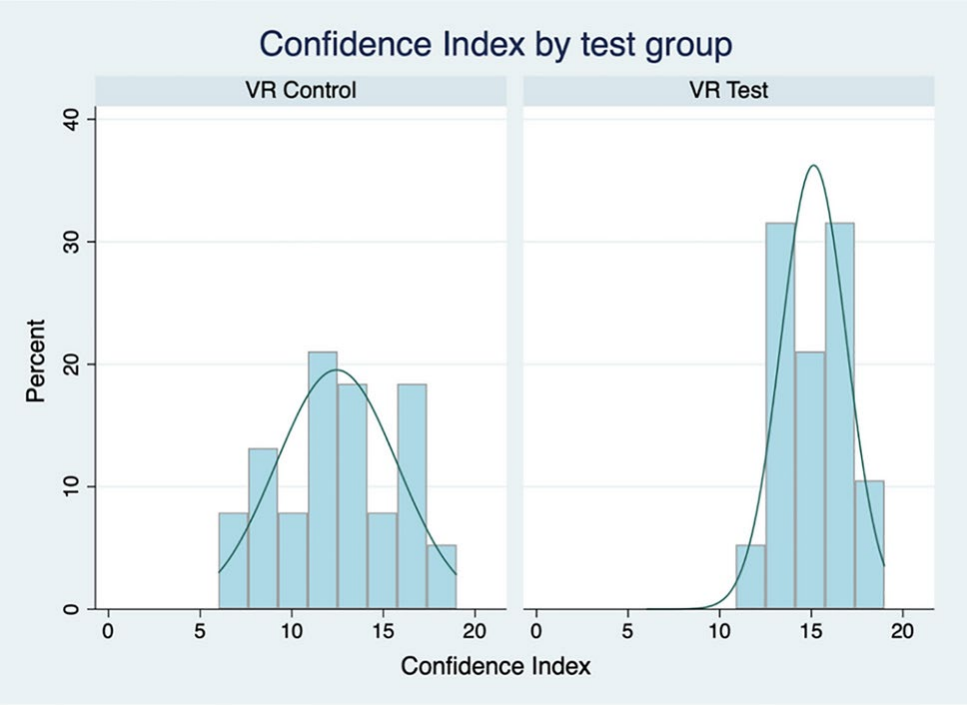
Distribution of learning confidence index by group

An additive confidence index (theoretical range 0–24) was created using the three confidence items (Cronbach’s alpha = 0.79). Figure 6 presents the confidence index distribution by group. As hypothesised, the mean difference between the two groups, at 2.68 (*p* < 0.01), reveals a significant difference between students in the VR-Test group and the VR-Control group, with the former being more confident than the latter in working in the laboratory environment following exposure to the VR-by-proxy lab simulation.

#### Differences in enjoyment

Our fourth hypothesis was that exposure to the simulated VR-by-proxy laboratory would improve students’ self-reported enjoyment of their learning. To test this, students in the two groups were asked different questions.

- Students in the VR-Test group were asked, “On a scale of 1–10, with 1 being not enjoyable and 10 being very enjoyable, where would you place the Molecular Methods online lab book (including the Virtual Reality (VR) lesson)?”
- Students in the VR-control group were asked, “On a scale of 1–10, with 1 being not enjoyable and 10 being very enjoyable, where would you place the Molecular Methods online lab book?”

**Fig. 7:**
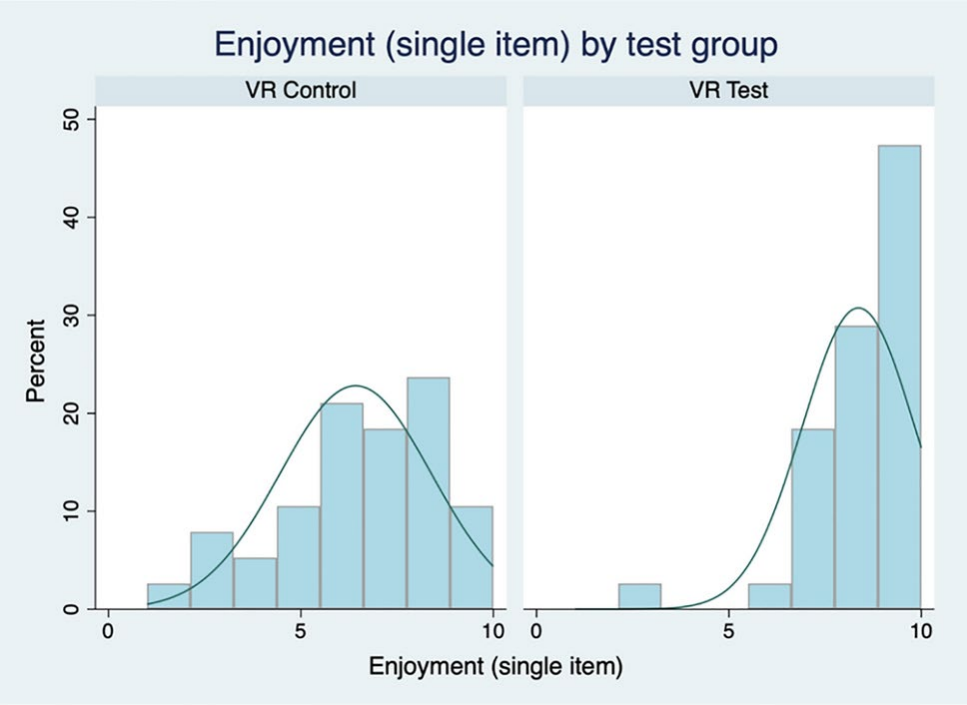
Distribution of enjoyment by group

Students in the VR-Test group on average rated their experience as 8.37 (s.d. = 0.24) on the 1–10 scale, whilst VR-Control group students rated the experience as 6.42 (s.d. = 0.32) on the same scale (Fig. 7). The groups are significantly different in their evaluations (diff = 1.95, *t* = 4.90); there is greater self-reported enjoyment with respect to the learning of the students who underwent the VR-by-proxy experience (hypothesis 4).

#### Controlling for individual characteristics

Despite the use of random assignment that placed subjects in the VR-Test and VR-Control groups, it is possible that students’ individual characteristics and previous experience with technology could influence their learning experience and/or confidence. To test for confounding influences, we ran OLS (Ordinary Least Squares) regression models predicting the learning experience and confidence indices as dependent variables with an indicator for subject test group (VR-Test group = 1; VR-Control = 0). Included in the right-hand side of the equations are control indicators for subject gender (female = 1), self-assessed social class (working class = 1) and whether one of the subject’s holds a university degree (parent degree = 1). We additionally control for subject’s previous experience in a laboratory environment (experience = 1) as those who report experience in a lab may have higher levels of confidence. Finally, students with previous experience with video gaming (never = 1; rarely = 2; occasionally = 3; great deal = 4) and/or virtual reality (never = 1; rarely = 2; occasionally = 3) might have felt more comfortable with the simulated VR experience. See S4 File for details of predictor variables.

Table 6 presents the results from the two regression models. Looking first at the model predicting *confidence*, students in the VR-Test group reported significantly higher levels of confidence. Further supporting hypothesis 3, holding all other variables in the model constant, those in the VR-Test group have an expected confidence almost four points higher than those in the VR-Control group (predicted value for VR-Test group = 19.14; No VR = 15.42).

**Table 6:** OLS regression of confidence and learning experience on test groups and control variables

	Confidence		Learning experience	
	b	(se)	b	(se)
VR-Test group	3.66**	(0.78)	8.41**	(1.36)
Female	0.54	(1.00)	0.23	(1.75)
Working class	− 0.27	(0.90)	1.69	(1.58)
Parents w/degree	1.08	(0.83)	2.60	(1.44)
Previous lab experience	0.36	(1.09)	1.73	(1.91)
Previous VR experience	2.59**	(0.88)	3.44*	(1.54)
Previous gaming experience	− 0.32	(0.50)	− 2.06*	(0.88)
Constant	12.09**	(1.49)	19.09**	(1.36)
N	74		74	
R^2^adj	.26		.40	

*p <.05 **p <.01

The only other significant predictor in the model, not surprisingly, is previous experience with VR, with those students saying they rarely use VR having a predicted confidence level about two points higher than those who have never used VR (18.69 versus 16.49) and those (very) few students who said they occasionally use VR being expected to be an additional 6 points higher (25.0).

Turning to the model predicting students’ reported *learning experience*, participants in the VR-Test group were about eight points higher on the learning experience index, with a predicted value of 29.44 (VR-Control group = 21.29). This provides substantial evidence in favour of our hypothesis that the Disease Diagnostic Laboratory VR lesson would enhance the student learning experience.

As with the model predicting confidence, we find that those students with previous exposure to VR were more likely to rate the learning experience more highly than those with no experience of VR. Interestingly, we find some evidence for the opposite effect with experience of video gaming. The model predicts, holding all other variables constant, that those with no experience playing video games would rate their learning experience about six points higher (27.0) than those with a great deal of gaming experience (21.0).

#### Nausea

As discussed above, one potential limitation to the use of virtual reality simulations in teaching—whether I-VR, D-VR or VR-by-proxy—could be the link between VR and nausea. As such, we hypothesised that those students who reported experiencing nausea during the VR-by-proxy laboratory would report a diminished experience. There is some evidence for this.

Students in both test groups were asked:

“Did you experience any feelings of nausea or dizziness during the lesson?”

Thirteen percent of students (5 students) in the VR-Test group reported feeling some sense of nausea/dizziness (compared to 3% (1 student) in the VR-Control group). Within the VR-Test group the students who reported feeling some sense of nausea or dizziness tended to report a less robust learning experience, by around 7 points on the learning experience index (23.4 versus 30.57 for those not experiencing a sense of nausea). However, interestingly, of the 5 students reporting a sense of nausea, 3 indicated they would recommend the VR learning method to other students (responding 6 or 7 on a 1–7 Likert scale).

#### Focus groups

Focus group transcripts were analysed using thematic analysis (Braun and Clarke 2021), based on a combination of deductive and inductive coding. Deductive codes were derived from the questions asked in the questionnaires and facilitator prompting questions to identify initial areas of interest that are relevant for this study. Inductive codes were then developed based on the ideas expressed by participants within the focus groups. These codes were used to identify broader themes and patterns within the data. Coding was undertaken manually by a researcher not involved in facilitating the focus groups in three stages.

First, *immersion*. This is a process of examining transcripts to identify a list of potential codes to guide more systematic codes in subsequent stages (Braun and Clarke 2021). This included noting general attitudes towards VR from group participants and any clear differences of opinion between participants which could skew the discussion in notable ways that would need to be considered when coding responses and interpreting the results. No consistent differences or biases were identified across each of the groups aside from the fact that the VR-Control group had been initially taught how to set up the qPCR experiment through a written online protocol (which the VR-Test group did not receive), whereas the VR-Test group were only shown this via VR-by-proxy, which had the potential to shape the content of their discussions. All focus groups took place after both Test and Control groups had completed the full VR-by-proxy experience.

Second, *selective coding*. This is a process of data reduction, to focus on items of interest for our research aims rather than coding all possible aspects of meaning within the transcript. Codes were generated in two ways. First, through coding individual participants’ responses to all facilitator questions, including follow-up questions. This allowed for direct comparison of participant responses to similar prompting questions. Second, through reviewing the list of potential codes from the immersion stage to identify relevant ideas expressed by participants. After removing some potential codes for not being relevant to this study, this list of codes was used to systematically code all transcripts.

Third, *identification of key themes and patterns*. This involved an iterative process of comparing data coded under each category, amongst participants in the same group, amongst participants in the same group classification, and comparisons between participants in the VR-test and VR-Control groups. During this process, four key themes emerged which capture the major topics of discussion within the focus groups:

- Understanding of aspects of the qPCR process;
- Familiarity with the laboratory;
- Comparisons between different teaching approaches;
- VR is fun.

#### Differences in confidence

In line with responses to the questionnaire, students in both the VR-Test and VR-Control groups made frequent and repeated claims that VR-by-proxy improved their levels of confidence about their learning. However, ‘confidence’ was understood in two different ways by participants: (1) confidence as ‘understanding’ the qPCR process; and (2) confidence as ‘familiarity’ with a laboratory setting.

##### Confidence as ‘understanding’ the qPCR process

In all focus groups, students were asked a prompting question about whether they felt more confident in their understanding of the qPCR process following exposure to the simulated VR laboratory. Most responses to this question indicated that students felt more confident. The most frequently stated reason was that VR-by-proxy allowed participants to ‘visualise’ the sequence of steps required to conduct the experiment. This was frequently accompanied by comments about how a ‘first-person’ perspective on the process allowed participants to feel like they were doing each of the steps themselves, to some degree. Some participants concentrated more on the visuality of the teaching method specifically, explicitly connecting their positive views of VR-by-proxy experience to their own self-identification as ‘visual learners’.

##### Confidence as ‘familiarity’ with a laboratory setting

Participants’ confidence in their understanding of the steps involved in the qPCR process did not extend to confidence about every aspect of that process. In responses to a prompting question about whether VR-by-proxy helped them to feel more confident about going into a laboratory setting and performing a qPCR, participants were more reticent. They offered fewer explicit claims about being able to perform every step of a qPCR experiment. Instead, participants in both the VR-Test and VR-Control groups were more likely to prefer the language of ‘familiarity’ when discussing the laboratory. They claimed that VR-by-proxy helped to familiarise them with what a laboratory looks like and what the process of conducting a qPCR experiment in this setting would look like, without describing themselves as confident in carrying out this experiment straight away.

Discussions about familiarity slightly differed between groups. Participants in VR-Test-B were not confident that they could work independently in a lab following the VR lesson, while participants in VR-Test-A and VR-Control-C were more confident as the VR lesson allowed them to visualise the steps of the experiment. VR-Control-A participants noted that although they would be more confident with conducting the experiment, this confidence did not extend to other aspects of the process such as data interpretation and analysis. This is consistent with our questionnaire response analysis in which VR-by-proxy did not result in a significant improvement in student confidence about analysing qPCR results independently.

#### Enjoyment and comparisons between teaching methods

Most students enjoyed the use of VR-by-proxy and compared it favourably to other teaching methods. Most comments were positive, with most participants noting how ‘fun’, ‘cool’, or ‘interesting’ it was to learn using VR. Individual participants in VR-Test-C and VR-Control-C spoke positively about VR-by-proxy teaching being the “closest we’ve been to a lab this year” and that this helped them to stay motivated or feel less isolated during lockdown.

Discussions about the relative merits of VR-by-proxy over other approaches took place in all focus groups, and often emerged unprompted as part of discussions about student confidence. Such comparisons took various forms, ranging from direct comparisons of teaching methods used in the course to more speculative discussions about the potential uses of VR-by-proxy in the future. Participants in the VR-Control groups tended to highlight the benefits of VR as one teaching method amongst others. They were more likely to give a ranked preferences order in which VR-by-proxy was ranked ‘higher’ than traditional teaching methods. Student used terms for describing VR-by-proxy included ‘immersive’, ‘engaging’ and ‘interactive’ although there was no unanimous agreement on these terms across the groups.

Participants in the VR-Test groups were more speculative about the potential uses of VR-by-proxy, noting how some elements of the other teaching methods (e.g. 2D simulations and graphs updating in real-time) could be incorporated into the VR demonstration to add detail. They also highlighted the value of using VR-by-proxy for revision. They didn’t tend to describe VR-by-proxy as immersive, engaging and interactive, and were also more forthright in saying they didn’t want to see VR replace physical labs.

Across all groups, the ‘complementarity’ of different teaching approaches was emphasised. For instance, Participant VR-Control-B-3 stated that:

I definitely think that [the] lab book and the VR complement each other really well to get the full understanding of everything, and to have one without the other you’re definitely going to miss a few things.

Many participants reemphasised earlier points they had made about VR-by-proxy helping them to visualise the process as a means of demonstrating the different steps in conducting a qPCR experiment. This was often distinguished from other aspects of the process which participants believed were important to learn such as ‘the theory’ behind the process and ‘what was happening at a molecular level’. When these distinctions were made, participants tended to speculate that teaching approaches other than VR-by-proxy would be more appropriate for learning about these elements of the process.

However, there was no consensus on how these teaching methods should be used together. While some participants placed more emphasis on using multiple teaching methods to teach the same thing, other participants spoke instead about the benefits of using different methods in sequence to teach different aspects of the process.

#### Nausea

Nausea was not a major theme in the focus groups. Nausea, motion sickness, dizziness or other related terms were only mentioned in direct response to prompting questions by the facilitators. Most comments focused on how the ‘jerkiness’ or ‘jumpiness’ of the video stream meant some participants thought they might experience nausea. A few participants experienced some nausea, but none considered this to be a barrier to their ability to take part in VR-by-proxy learning. However, participants 1A-1 and 2A-4 argued that if the lesson had been longer than 30 min they expect that they would have experienced nausea more acutely.

## Discussion

The COVID-19 pandemic resulted in a significant switch to online and blended learning with the student at the centre. Implementing a VR-by-proxy laboratory experience into our course allowed the students to view practical, hands-on techniques during a time when social distancing requirements made in-person practical teaching impossible. While VR is generally not considered by educators to be a replacement for traditional teaching modalities, there is a growing body of evidence that VR may enhance the student experience (Alhalabi 2016; Loup et al. 2016; Krokos et al. 2019; Klingenberg et al. 2020). VR-by-proxy is a novel method of using this technology, potentially enabling widespread use of VR technology, due to the many benefits it brings compared to I-VR, such as costs, environmental impacts and space saving.

In line with hypothesis 1, VR-by-proxy did not significantly enhance student learning in relation to their theoretical knowledge. This is in agreement with other recent studies that similarly showed that the direct impact on learning outcomes was limited (Coban et al. 2022; Matovu et al. 2023), although; findings across the field remain in disagreement on this point (Concannon et al. 2019; Radianti et al. 2020; Tsirulnikov et al. 2023). Indeed, Clark suggested that changing media should make no difference to student outcomes if all other factors are held constant (Clark 1983). However, in line with our initial hypotheses 2, 3 and 4, we found that exposure to a VR-by-proxy laboratory had a positive effect on students’ self-reported learning experience, confidence, and enjoyment of learning. These results hold after controlling for individual characteristics.

In our focus groups, confidence was understood in two ways by participants: firstly, as ‘understanding’ of the various steps involved in conducting a qPCR experiment and secondly as a more general ‘familiarity’ with the laboratory setting and equipment. While most participants enjoyed the experience, different views were apparent between the VR-Test and Control groups about how ‘immersive’, ‘engaging’ and ‘interactive’ they perceived the VR-by-proxy experience to be and what role it should play alongside other teaching methods. For example, our results demonstrated that many participants found the VR-by-proxy experience helped them visualise the different steps involved in setting up a qPCR experiment. However, although the Disease Diagnostic Laboratory lesson does not demonstrate the molecular theory behind the qPCR technique, or how to analyse qPCR results, students suggested that it would not be suitable to cover this aspect. Analysis of qPCR results were not included in the simulation, so this finding was to be expected. Students felt alternative approaches would be more beneficial for learning about the molecular theory behind this technique. This finding is of interest because previous studies have shown that I-VR and computer simulations do benefit learning of complex molecular biology concepts (Reen et al. 2021, 2022; Sun and Zhao 2023). Whether students failed to imagine the way VR may be used to teach molecular theory is unknown.

Students in the VR-Control group initially worked through the Moodle lab book, supplemented by a written protocol, thus they initially completed the lesson using only traditional methods prior to the VR-by-proxy experience. The VR-Test group experienced the VR-by-proxy as an alternative to receiving the written protocol. It could be speculated that the positive enjoyment scores of the VR-Test group who received the VR-by-proxy experience, compared to the VR-Control group who did not, are due to the novelty of the experience within their teaching programmes. This in turn may impact a student’s ability to process information e.g. due to distractions (Hamilton et al. 2021). Further research would be required to examine alternative approaches of integrating VR-by-proxy to facilitate engagement in deeper learning. This is likely to require assessment of VR-by-proxy as part of a suite of teaching methods, and investigation of different ways of combining and sequencing them, to establish which have a positive impact on students’ learning experience, confidence, and learning in relation to theoretical knowledge. This should be done in a way that supports students to build on their previous knowledge, as well as aligning with course learning goals.

In our study, the use of a VR simulation, delivered by-proxy, mitigated the lack of in-person laboratory-based teaching by providing our students with an immersive and realistic experience whilst they worked remotely. Educators are acutely aware that post-COVID, many students are experiencing anxiety and stress when returning to campus (Rashid et al. 2022; Basheti et al. 2023). This study shows that VR-by-proxy improves students’ confidence and learning experience, and therefore offers an opportunity to support hybrid flexible learning strategies that are often a preferred method of learning post-pandemic (Guppy et al. 2022).

Our findings indicate that some students express learning preferences. This may explain why VR-by-proxy has some positive effects on students’ self-reported learning experience, confidence and enjoyment. For example, students who purport to be visual or aural learners may benefit more significantly from VR-by-proxy than self-described kinaesthetic learners. Perhaps unsurprisingly, those students familiar with the use of VR technology may also have benefited more from the experience. It is important to note, however, that matching instruction to students’ self-reported learning preferences is unlikely to result in enhanced knowledge attainment. There is extensive literature within educational and cognitive psychology which highlights the lack of evidence that matching instruction to learning styles enhances knowledge attainment (Willingham et al. 2015). Our findings do not offer any evidence to support this practice.

Innovative learning technologies require an inclusive approach to their development and delivery. Our findings showed that the few students who reported experiencing nausea during the VR-by-proxy laboratory reported a diminished learning experience. This is in line with reports in the current literature according to which users of VR technology can experience nausea similar to motion sickness (Stanney et al. 2020). Sex-specific effects have been noted in cybersickness when using I-VR, with suggestions that these differences depend on the task being performed (Curry et al. 2020). Although the students in this study said the feeling of nausea did not prevent them from completing the lesson, cybersickness is an undesirable side effect of the technology, and should be considered when developing VR for teaching, particularly if this may lead to discrimination of certain cohorts. Negative health impacts, such as eye strain, vestibular symptoms or symptoms associated with poorly adjusted hardware (e.g. headaches or neck pain), should be monitored when evaluating immersive tools as a teaching resource and solutions should be adopted within the technology and content design to ensure equity (Heilemann et al. 2021; Skulmowski 2023).

### Study limitations

The main limitation of the study is the sample size, as during the COVID-19 pandemic, recruitment of students was online and challenging. For future studies, a greater number of students would be beneficial. The Disease Diagnostics VR-by-proxy simulation being used was fixed-pace, as the instructor was working through the simulation, narrating the lesson. This was a limitation of the approach, as students could not pause or replay when viewing. This was mitigated by offering a recorded version of the simulation following the synchronous lesson, however alternative approaches such as asking the group of students viewing the simulation to request for the simulation to be replayed could be used.

### Future work and usage

VR-by-proxy is a novel teaching approach that has the potential to engage students and enhance the learning experience, and this study adds to the growing body of literature that supports the finding that immersive educational tools have a positive impact on students. It may be particularly effective in countries with limited resources and less advanced laboratory infrastructure (Ray and Srivastava 2020). This method supports students learning practical skills while reducing the need for extensive laboratory exposure, and multiple VR headsets, making it a sustainable and cost-effective alternative. In addition, with increasing numbers of students in HE, space on campus comes at a high premium, particularly laboratory space and the ability to provide students with adequate training in key practical skills required for employment. Using the technology could enable educators to embed VR into the curriculum at relatively low cost, with high gains. In the future, a comparative study looking at VR-by-proxy versus traditional VR headset use (I-VR) within an HE context, would be beneficial, as would further investigations into the nausea and sex-effects of VR, to ensure inclusive curriculum design. Specific methodologies such as pre and post questionnaires, focus groups and simulation analytics could be used to further examine such areas of research.

## Supplementary Information

Below is the link to the electronic supplementary material.

Supplementary file1 (DOCX 42 KB)

Supplementary file2 (DOCX 24 KB)

Supplementary file3 (DOCX 25 KB)

Supplementary file4 (DOCX 26 KB)

## Data Availability

All data will be fully available online on publication of the paper.

## Footnotes

1 We made use of Pearson’s Chi-squared tests rather than Fisher’s exact test as some of the cells in the 2X2 tables exceeded the recommended upper count limit of 20.

## References

[CR1] Al-Elq AH (2010) Simulation-based medical teaching and learning. J Fam Community Med 17(1):35–40 10.4103/1319-1683.68787 PMC3195067 22022669

[CR2] Alhalabi W (2016) Virtual reality systems enhance students’ achievements in engineering education. Behav Inf Technol 35(11):919–925

[CR3] Basheti IA, Assaraira TY, Obeidat NM, Al-Abed Al-Haq F, Refai M (2023) Assessing anxiety and depression among students post-COVID-19: exploring associating factors. Psychol Res Behav Manag 16:1797–1810 37201174 10.2147/PRBM.S409632 PMC10187645

[CR4] Braun V, Clarke V (2021) Thematic analysis. American Psychological Association

[CR5] Burns T, Köste F (2016) Governing education in a complex world. OECD Publishing, Paris

[CR6] Chang E, Kim HT, Yoo B (2020) Virtual reality sickness: a review of causes and measurements. Int J Hum–comput Interact 36(17):1658–1682

[CR7] Clark RE (1983) Reconsidering research on learning from media. Rev Educ Res 53(4):445–459

[CR8] Coban M, Bolat YI, Goksu I (2022) The potential of immersive virtual reality to enhance learning: a meta-analysis. Educ Res Rev 36:100452

[CR9] Concannon BJ, Esmail S, Roduta Roberts M (2019) Head-mounted display virtual reality in post-secondary education and skill training. In: Frontiers in education. Frontiers Media SA

[CR10] Conrad M, Kablitz D, Schumann S (2024) Learning effectiveness of immersive virtual reality in education and training: a systematic review of findings. Comput Educ X Real 4:100053

[CR11] Curry C, Li R, Peterson N, Stoffregen TA (2020) Cybersickness in virtual reality head-mounted displays: examining the influence of sex differences and vehicle control. Int J Hum–comput Interact 36(12):1161–1167

[CR12] Faria AJ, Wellington WJ (2004) A survey of simulation game users, former-users, and never-users. Simul Gaming 35(2):178–207

[CR13] Guppy N, Verpoorten D, Boud D, Lin L, Tai J, Bartolic S (2022) The post-COVID-19 future of digital learning in higher education: views from educators, students, and other professionals in six countries. Br J Edu Technol 53(6):1750–1765

[CR14] Hamilton D, McKechnie J, Edgerton E, Wilson C (2021) Immersive virtual reality as a pedagogical tool in education: a systematic literature review of quantitative learning outcomes and experimental design. J Comput Educ 8(1):1–32

[CR15] Hays RT, Singer MJ (1989) Simulation fidelity in training system design: bridging the gap between reality and training. Springer-Verlag Publishing, New York

[CR16] Heilemann F, Zimmermann G, Münster P (2021) Accessibility guidelines for vr games—a comparison and synthesis of a comprehensive set. Front Virtual Real 2:697504

[CR17] Hodgson E, Bachmann ER, Vincent D, Zmuda M, Waller D, Calusdian J (2015) WeaVR: a self-contained and wearable immersive virtual environment simulation system. Behav Res Methods 47(1):296–307 24737097 10.3758/s13428-014-0463-1

[CR18] Jensen L, Konradsen F (2018) A review of the use of virtual reality head-mounted displays in education and training. Educ Inf Technol 23(4):1515–1529

[CR19] Johns G (2006) The essential impact of context on organizational behavior. The Academy of Management Review, 31(2):386–408. 10.2307/20159208

[CR20] Jones N (2018) Simulated labs are booming. Nature 562(7725):S5-s7 30283122 10.1038/d41586-018-06831-1

[CR21] Klingenberg S, Jørgensen MLM, Dandanell G, Skriver K, Mottelson A, Makransky G (2020) Investigating the effect of teaching as a generative learning strategy when learning through desktop and immersive VR: a media and methods experiment. Br J Edu Technol 51(6):2115–2138

[CR22] Krosnick JA, Fabrigar LR (1997) Designing rating scales for effective measurement in surveys. Survey measurement and process quality. 141–164

[CR23] Krosnick JA (1999) Survey research. Annu Rev Psychol 50:537–567. 10.1146/annurev.psych.50.1.537 15012463

[CR24] Krosnick JA, Presser S (2010) Handbook of survey research: Question and questionnaire design. Handbook of survey research: Question and Questionnaire Design 2:264–313

[CR25] Krokos E, Plaisant C, Varshney A (2019) Virtual memory palaces: immersion aids recall. Virtual Real 23:1–15

[CR26] Loup G, Serna A, Iksal S, George S (2016) Immersion and persistence: improving learners’ engagement in authentic learning situations. Springer International Publishing, Cham

[CR27] Makransky G, Lilleholt L (2018) A structural equation modeling investigation of the emotional value of immersive virtual reality in education. Educ Tech Res Dev 66(5):1141–1164

[CR28] Makransky G, Borre-Gude S, Mayer RE (2019) Motivational and cognitive benefits of training in immersive virtual reality based on multiple assessments. J Comput Assist Learn 35(6):691–707

[CR29] Makransky G, Mayer R, Nøremølle A, Cordoba AL, Wandall J, Bonde M (2020) Investigating the feasibility of using assessment and explanatory feedback in desktop virtual reality simulations. Educ Tech Res Dev 68(1):293–317

[CR30] Markowitz DM, Laha R, Perone BP, Pea RD, Bailenson JN (2018) Immersive virtual reality field trips facilitate learning about climate change. Front Psychol 9:2364 30555387 10.3389/fpsyg.2018.02364 PMC6284182

[CR31] Matovu H, Ungu DAK, Won M, Tsai C-C, Treagust DF, Mocerino M, Tasker R (2023) Immersive virtual reality for science learning: design, implementation, and evaluation. Stud Sci Educ 59(2):205–244

[CR32] McCauley ME, Sharkey TJ (1992) Cybersickness: perception of self-motion in virtual environments. Presence Teleoperators Virtual Environ 1(3):311–318

[CR33] Osterman KF (2000) Students’ need for belonging in the school community. Rev Educ Res 70(3):323–367

[CR34] Ott M, Freina L (2015) A literature review on immersive virtual reality in education: state of the art and perspectives. In: 11th international conference elearning and software for education

[CR35] Perry S, Bridges SM, Burrow MF (2015) A review of the use of simulation in dental education. Simul Healthc 10(1):31–37 25574865 10.1097/SIH.0000000000000059

[CR36] Pregowska A, Osial M, Gajda A (2023) What will the education of the future look like? How have metaverse and extended reality affected the higher education systems? Metaverse Basic Appl Res 3:57

[CR37] Radianti J, Majchrzak TA, Fromm J, Wohlgenannt I (2020) A systematic review of immersive virtual reality applications for higher education: design elements, lessons learned, and research agenda. Comput Educ 147:103778

[CR38] Rashid S, Shaikh S, Mardini L, Saad FS (2022) Back to school: COVID-19 post-lockdown classroom anxiety. Educ Sci 12(11):800

[CR39] Ray S, Srivastava S (2020) Virtualization of science education: a lesson from the COVID-19 pandemic. J Proteins Proteom 11(2):77–80 33132627 10.1007/s42485-020-00038-7 PMC7261257

[CR40] Reen FJ, Jump O, McSharry BP, Morgan J, Murphy D, O’Leary N, O’Mahony B, Scallan M, Supple B (2021) The use of virtual reality in the teaching of challenging concepts in virology, cell culture and molecular biology. Front Virtual Real 2:670909

[CR41] Reen FJ, Jump O, McEvoy G, McSharry BP, Morgan J, Murphy D, O’Leary N, O’Mahony B, Scallan M, Walsh C, Supple B (2022) Developing student codesigned immersive virtual reality simulations for teaching of challenging concepts in molecular and cellular biology. FEMS Microbiol Lett 369(1):fnac051 35671125 10.1093/femsle/fnac051 PMC9279883

[CR42] Ryan GV, Callaghan S, Rafferty A, Higgins MF, Mangina E, McAuliffe F (2022) Learning outcomes of immersive technologies in health care student education: systematic review of the literature. J Med Internet Res 24(2):e30082 35103607 10.2196/30082 PMC8848248

[CR43] Salzman MC, Dede C, Loftin RB, Chen J (1999) A model for understanding how virtual reality aids complex conceptual learning. Presence Teleoperators Virtual Environ 8(3):293–316

[CR44] Saris WE, Gallhofer I (2007) Estimation of the effects of measurement characteristics on the quality of survey questions. Surv Res Methods. 1(1):29–43. 10.18148/srm/2007.v1i1.49

[CR45] Saris W, Revilla M, Krosnick JA, Shaeffer EM (2010) Comparing questions with Agree/Disagree response options to questions with Item-Specific response options. Surv Res Methods. 4(1):61–79. 10.18148/srm/2010.v4i1.2682

[CR46] Skulmowski A (2023) Ethical issues of educational virtual reality. Comput Educ X Real 2:100023

[CR47] Stanney K, Lawson BD, Rokers B, Dennison M, Fidopiastis C, Stoffregen T, Weech S, Fulvio JM (2020) Identifying causes of and solutions for cybersickness in immersive technology: reformulation of a research and development agenda. Int J Hum–comput Interact 36(19):1783–1803

[CR48] Sun T, Zhao Z (2023) Teaching dynamic mechanisms in signaling pathways using computational simulations. Educ Chem Eng 42:20–30

[CR49] Tabatabai S (2020) COVID-19 impact and virtual medical education. J Adv Med Educ Prof 8(3):140–143 32802908 10.30476/jamp.2020.86070.1213 PMC7395196

[CR50] Toquero CM (2020) Emergency remote education experiment amid COVID-19 pandemic. IJERI Int J Educ Res Innov 15:162–176

[CR51] Tsirulnikov D, Suart C, Abdullah R, Vulcu F, Mullarkey CE (2023) Game on: immersive virtual laboratory simulation improves student learning outcomes & motivation. FEBS Open Bio 13(3):396–407 10.1002/2211-5463.13567 PMC9989934 36723273

[CR52] Weech S, Kenny S, Barnett-Cowan M (2019) Presence and cybersickness in virtual reality are negatively related: a review. Front Psychol 10:158 30778320 10.3389/fpsyg.2019.00158 PMC6369189

[CR53] Willingham DT, Hughes EM, Dobolyi DG (2015) The scientific status of learning styles theories. Teach Psychol 42(3):266–271

[CR54] Wyk Ev, Villiers Rd (2009) Virtual reality training applications for the mining industry. In: Proceedings of the 6th international conference on computer graphics, virtual reality, visualisation and interaction in Africa. Pretoria, South Africa, Association for Computing Machinery, pp 53–63

[CR55] Zawacki-Richter O, Latchem C (2018) Exploring four decades of research in computers & education. Comput Educ 122:136–152

[CR56] Zhao J, Xu X, Jiang H, Ding Y (2020) The effectiveness of virtual reality-based technology on anatomy teaching: a meta-analysis of randomized controlled studies. BMC Med Educ 20(1):127 32334594 10.1186/s12909-020-1994-z PMC7183109

