# Supplementary material for: Experiential learning through virtual reality by-proxy
Source: Virtual Real. 2025 Feb 8;29(1):38. doi: 10.1007/s10055-025-01106-3 (PMC11906506; doi:10.1007/s10055-025-01106-3)
Supplement: Supplementary file 1 — Supplementary file1 (DOCX 42 KB) [file 10055_2025_1106_MOESM1_ESM.docx]

**Questionnaire 1**

1. Do you have any experience in a molecular research diagnostics lab? Y/N
2. How much experience with Virtual Reality (VR) technology do you have?

I have a great deal of experience with VR technology.

I frequently use VR.

I occasionally use VR.

I rarely use VR.

I have never used VR.

1. If you have used Virtual Reality (VR) technology before, can you tell us what equipment you have used? Please select all that apply.

Google Cardboard

Samsung Gear VR

Other version of VR which involved a mobile phone in a box

Oculus Go

Oculus Quest/Quest 2

Google Daydream

HTC Vive Focus

Another version of VR which was standalone (no PC or console was required to run the VR system)

Playstation VR

Oculus Rift/Rift S

HTC Vive/Vive Pro

HTC ViveCosmos

Pico Neo/Neo 2

Lenovo/Dell/HP

Samsung Odyssey

Other version of VR that was Tethered (the VR system had to be connected to a PC or console to run)

1. How much experience with video gaming do you have?

I have a great deal of experience with video games.

I occasionally play video games.

I rarely play video games.

I never play video games.

1. How comfortable are you using computer technology beyond basic word-processing, web-browsing and playing video games?

I am very comfortable.

I am moderately comfortable.

I am moderately uncomfortable.

I am very uncomfortable.

1. What is your gender?

Male

Female

Non-binary

Gender-fluid

Other

Prefer not to say

1. What is your degree group?

Anatomy

Physiology

Pharmacology

Human Biology

Neuroscience

Microbiology/Immunology

Zoology/Marine and Freshwater Biology

Genetics/MCB/Biochemistry

1. If you had to choose, what is your social class?

Upper class

Upper middle class

Middle class

Lower middle class

Upper working class

Working class

1. Do any of your parents have a university degree?

Yes

No

Not sure

1. How confident would you feel if asked to perform a qPCR experiment independently?

Extremely confident

Confident

Somewhat confident

Neutral

Somewhat not confident

Not confident

Not confident at all

1. How confident would you be performing a qPCR experiment independently?

Extremely confident

Confident

Somewhat confident

Neutral

Somewhat not confident

Not confident

Not confident at all

1. How confident do you feel about analysing qPCR results independently?

Extremely confident

Confident

Somewhat confident

Neutral

Somewhat not confident

Not confident

Not confident at all

1. How confident would you feel working aseptically using a lamina flow hood?

Extremely confident

Confident

Somewhat confident

Neutral

Somewhat not confident

Not confident

Not confident at all

1. Place these stages of setting up a pipette in the correct order:
   1. Push plunger to the first stop

b. Push plunger to the second stop

c. Dispense liquid

d. Aspirate liquid

e. Pick up a tip

f. Place tip into liquid

g. Eject tip

1. What is the function of SYBR green in a qPCR?
   1. To detect DNA bands during gel electrophoresis
   2. To bind to double stranded DNA and fluoresce
   3. To bind to single stranded DNA and fluoresce
   4. To allow the polymerase to add nucleotides
   5. I don’t know
2. Why is it important to set up a Master Mix?
   1. To reduce pipetting error
   2. To minimise the risk of contamination
   3. It is convenient and saves time
   4. All of the above
   5. I don’t know
3. What steps can we take to prevent contamination when setting up a qPCR?
   1. Clean surfaces and equipment before and after with 70% ethanol
   2. Setting up each stage on the bench
   3. Handling multiple samples simultaneously
   4. I don’t know
4. What are the key advantages to using qPCR over traditional PCR - tick all the apply

Sensitive

Rapid

High through-put

Cheaper

Quantification of RNA

I am unsure

1. The higher the cycle threshold (Ct-value) the greater the starting quantity of DNA present in the sample. This statement is:

True

False

I don't know

**Questionnaire 2a**

1. Were you able to watch the video of the Virtual Reality lesson? Y/N
2. Please tell us on what type of device you viewed the Virtual Reality lesson.

A computer monitor

A laptop

A mobile phone

A tablet

Other

1. How confident are you in your understanding of quantitative PCR (qPCR)?

Extremely confident

Confident

Somewhat confident

Neutral

Somewhat not confident

Not confident

Not confident at all

1. How confident would you be performing a qPCR experiment independently?

Extremely confident

Confident

Somewhat confident

Neutral

Somewhat not confident

Not confident

Not confident at all

1. How confident do you feel about analysing qPCR results independently?

Extremely confident

Confident

Somewhat confident

Neutral

Somewhat not confident

Not confident

Not confident at all

1. How confident would you feel working aseptically using a lamina flow hood?

Extremely confident

Confident

Somewhat confident

Neutral

Somewhat not confident

Not confident

Not confident at all

1. Place these stages of setting up a pipette in the correct order:
   1. Push plunger to the first stop

b. Push plunger to the second stop

c. Dispense liquid

d. Aspirate liquid

e. Pick up a tip

f. Place tip into liquid

g. Eject tip

1. What is the function of SYBR green in a qPCR?
   1. To detect DNA bands during gel electrophoresis
   2. To bind to double stranded DNA and fluoresce
   3. To bind to single stranded DNA and fluoresce
   4. To allow the polymerase to add nucleotides
   5. I don’t know
2. Why is it important to set up a Master Mix?
   1. To reduce pipetting error
   2. To minimise the risk of contamination
   3. It is convenient and saves time
   4. All of the above
   5. I don’t know
3. What steps can we take to prevent contamination when setting up a qPCR?
   1. Clean surfaces and equipment before and after with 70% ethanol
   2. Setting up each stage on the bench
   3. Handling multiple samples simultaneously
   4. I don’t know
4. What are the key advantages to using qPCR over traditional PCR - tick all the apply

Sensitive

Rapid

High through-put

Cheaper

Quantification of RNA

I am unsure

1. The higher the cycle threshold (Ct-value) the greater the starting quantity of DNA present in the sample. This statement is:

True

False

I don't know

1. To what extent did you understand the qPCR process demonstrated in Virtual Reality (VR)?

Completely understood

Understood

Somewhat understood

Neutral

Somewhat did not understand

Did not understand

Completely did not understand

1. How present did you feel in the Virtual Reality VR qPCR scenario that you were engaging with?

Fully present

Present

Somewhat present

Neutral

Somewhat not present

Not present

Fully not present

1. To what extent did you feel like you were in the lab during the lesson?

I fully felt like I was in the lab

I felt like I was in the lab to a considerable degree

I somewhat felt like I was in the lab

I felt neutral about whether I was in the lab or not in the lab

I somewhat felt like I was not in the lab

I felt like I wasn't in the lab to a considerable degree

I fully felt like I was not in the lab

1. To what extent did you feel like you were being demonstrated to live in a lab environment?

I fully felt like I was being demonstrated to live in a lab environment

I felt like I was being demonstrated to live in a lab environment to a considerable extent

I somewhat felt like I was being demonstrated to live in a lab environment

I felt neutral

I somewhat felt like I was not being demonstrated to live in a lab environment

I felt like I was not being demonstrated to live in a lab environment to a considerable extent

I fully felt like I was not being demonstrated to live in a lab environment

1. To what extent did the teaching method improve your understanding of qPCR?

The teaching method had a strongly positive effect on my understanding of qPCR

The teaching method had a positive effect on my understanding of qPCR

The teaching method had a somewhat positive effect on my understanding of qPCR

The teaching method had no effect on my understanding of qPCR

The teaching method had a somewhat negative effect on my understanding of qPCR

The teaching method had a negative effect on my understanding of qPCR

The teaching method had a strongly negative effect on my understanding of qPCR

1. How likely would you be to recommend this method of learning to another student?

Very likely

Likely

Somewhat likely

Neither likely nor unlikely

Somewhat unlikely

Unlikely

Very unlikely

1. On a scale of 1-10, with 1 being not enjoyable and 10 being very enjoyable, where would you place the Molecular Methods online lab book (including the Virtual Reality (VR) lesson)?

10 - Very enjoyable

9

8

7

6

5

4

3

2

1 - Not enjoyable

1. How would you describe your experience of the lesson (including the Virtual Reality (VR) lesson)? [open text box]
2. Did you experience any feelings of nausea or dizziness during the lesson?

Yes

No

Not sure

1. On a scale of 1-10, with 1 being very nauseous and 10 being not at all nauseous, how nauseous did you feel during the lesson?

10 - Not at all nauseous

9

8

7

6

5

4

3

2

1 – Very nauseous

1. Did you experience any negative side-effects during the lesson? If so, please explain what these were. [open text box]
2. Do you have any additional comments on Virtual Reality (VR) teaching? [open text box]

**Questionnaire 2b**

1. How confident are you in your understanding of quantitative PCR (qPCR)?

Extremely confident

Confident

Somewhat confident

Neutral

Somewhat not confident

Not confident

Not confident at all

1. How confident would you be performing a qPCR experiment independently?

Extremely confident

Confident

Somewhat confident

Neutral

Somewhat not confident

Not confident

Not confident at all

1. How confident do you feel about analysing qPCR results independently?

Extremely confident

Confident

Somewhat confident

Neutral

Somewhat not confident

Not confident

Not confident at all

1. How confident would you feel working aseptically using a lamina flow hood?

Extremely confident

Confident

Somewhat confident

Neutral

Somewhat not confident

Not confident

Not confident at all

1. Place these stages of setting up a pipette in the correct order:
   1. Push plunger to the first stop

b. Push plunger to the second stop

c. Dispense liquid

d. Aspirate liquid

e. Pick up a tip

f. Place tip into liquid

g. Eject tip

1. What is the function of SYBR green in a qPCR?
   1. To detect DNA bands during gel electrophoresis
   2. To bind to double stranded DNA and fluoresce
   3. To bind to single stranded DNA and fluoresce
   4. To allow the polymerase to add nucleotides
   5. I don’t know
2. Why is it important to set up a Master Mix?
   1. To reduce pipetting error
   2. To minimise the risk of contamination
   3. It is convenient and saves time
   4. All of the above
   5. I don’t know
3. What steps can we take to prevent contamination when setting up a qPCR?
   1. Clean surfaces and equipment before and after with 70% ethanol
   2. Setting up each stage on the bench
   3. Handling multiple samples simultaneously
   4. I don’t know
4. What are the key advantages to using qPCR over traditional PCR - tick all the apply

Sensitive

Rapid

High through-put

Cheaper

Quantification of RNA

I am unsure

1. The higher the cycle threshold (Ct-value) the greater the starting quantity of DNA present in the sample. This statement is:

True

False

I don't know

1. To what extent did you understand the qPCR process demonstrated by reading through the document/via the qPCR 2D animated Learning Science demonstrations?

I was completely able to understand the process

I was able to understand the process to a considerable degree

I was somewhat able to understand the process

Neutral

I was somewhat unable to understand the process

I was unable to understand the process to a considerable degree

I was completely unable to understand the process

1. How present did you feel in the online lab manual qPCR scenario that you were engaging with?

Fully present

Present

Somewhat present

Neutral

Somewhat not present

Not present

Fully not present

1. To what extent did you feel like you were in the lab during the lesson?

I fully felt like I was in the lab

I felt like I was in the lab to a considerable degree

I somewhat felt like I was in the lab

I felt neutral about whether I was in the lab or not in the lab

I somewhat felt like I was not in the lab

I felt like I wasn't in the lab to a considerable degree

I fully felt like I was not in the lab

1. To what extent did you feel like you were being demonstrated to live in a lab environment?

I fully felt like I was being demonstrated to live in a lab environment

I felt like I was being demonstrated to live in a lab environment to a considerable extent

I somewhat felt like I was being demonstrated to live in a lab environment

I felt neutral

I somewhat felt like I was not being demonstrated to live in a lab environment

I felt like I was not being demonstrated to live in a lab environment to a considerable extent

I fully felt like I was not being demonstrated to live in a lab environment

1. To what extent did the teaching method improve your understanding of qPCR?

The teaching method had a strongly positive effect on my understanding of qPCR

The teaching method had a positive effect on my understanding of qPCR

The teaching method had a somewhat positive effect on my understanding of qPCR

The teaching method had no effect on my understanding of qPCR

The teaching method had a somewhat negative effect on my understanding of qPCR

The teaching method had a negative effect on my understanding of qPCR

The teaching method had a strongly negative effect on my understanding of qPCR

1. How likely would you be to recommend this method of learning to another student?

Very likely

Likely

Somewhat likely

Neither likely nor unlikely

Somewhat unlikely

Unlikely

Very unlikely

1. On a scale of 1-10, with 1 being not enjoyable and 10 being very enjoyable, where would you place the Molecular Methods online lab book?

10 - Very enjoyable

9

8

7

6

5

4

3

2

1 - Not enjoyable

1. How would you describe your experience of the lesson? [open text box]
2. Did you experience any feelings of nausea or dizziness during the lesson?

Yes

No

Not sure

1. On a scale of 1-10, with 1 being very nauseous and 10 being not at all nauseous, how nauseous did you feel during the lesson?

10 - Not at all nauseous

9

8

7

6

5

4

3

2

1 – Very nauseous

1. Did you experience any negative side-effects during the lesson? If so, please explain what these were. [open text box]

**Questionnaire 3**

1. Were you able to view the Virtual Reality lesson? Y/N
2. Please tell us on what type of device you viewed the Virtual Reality lesson.

A computer monitor

A laptop

A mobile phone

A tablet

Other

1. Was the lesson with the VR experience more enjoyable than the lesson without it? Explain your answer. [open text box]
2. Do you think you learned more in the VR lesson than the lesson without VR?

Yes

No

I’m not sure

1. Did you feel more like you were being demonstrated io live in a lab environment in the VR lesson than in the lesson without it?

Yes

No

I’m not sure

1. Which method of learning are you more likely to recommend (VR or no VR)? [open text box]
2. Did you feel any difference in terms of nausea in the VR lesson compared to the lab book lesson?

Yes- more nausea with VR

Yes- less nausea with VR

No difference

I’m not sure

1. Do you have any additional comments on Virtual Reality (VR) teaching? [open text box]
