# Supplementary material for: Experiential learning through virtual reality by-proxy
Source: Virtual Real. 2025 Feb 8;29(1):38. doi: 10.1007/s10055-025-01106-3 (PMC11906506; doi:10.1007/s10055-025-01106-3)
Supplement: Supplementary file 2 — Supplementary file2 (DOCX 24 KB) [file 10055_2025_1106_MOESM2_ESM.docx]

**Focus group questions**

- Background question: How much experience of VR do you have?
- We are curious about how much the VR demonstration helped you to understand the qPCR process. Do you feel like the VR demonstration helped you understand what to do in the lab? How so?
- After watching the qPCR VR demonstration do you think you would be more confident working in a real lab? How so?
- Comparing the qPCR VR demonstration to, say, the workbook exercises, in what ways was the VR demonstration better or worse than the workbook?
- Was the VR demonstration enjoyable and interesting?
