# Supplementary material for: Experiential learning through virtual reality by-proxy
Source: Virtual Real. 2025 Feb 8;29(1):38. doi: 10.1007/s10055-025-01106-3 (PMC11906506; doi:10.1007/s10055-025-01106-3)
Supplement: Supplementary file 3 — Supplementary file3 (DOCX 25 KB) [file 10055_2025_1106_MOESM3_ESM.docx]

**Supplementary File 3 Hyperlinks**

Disease Diagnostic Laboratory voice-over walk through:

<https://www.edify.ac/industry-solutions/biosciences-edify-training-solution-disease-diagnostics-laboratory>

2D ‘Learning Science’ qPCR simulation:

<https://www.learnsci.com/products/labsims>
