# Supplementary material for: Experiential learning through virtual reality by-proxy
Source: Virtual Real. 2025 Feb 8;29(1):38. doi: 10.1007/s10055-025-01106-3 (PMC11906506; doi:10.1007/s10055-025-01106-3)
Supplement: Supplementary file 4 — Supplementary file4 (DOCX 26 KB) [file 10055_2025_1106_MOESM4_ESM.docx]

Supplemental Materials 3 (S3): Descriptive statistics for variables included in OLS regression models

**Variable: Female**

|  | Freq. | Percent |
| --- | --- | --- |
| Men (0) | 68 | 80.00 |
| Non-Binary (0.5) | 1 | 1.18 |
| Female (1) | 16 | 18.82 |
| N | 85 |  |

**Variable: Working Class**

|  | Freq. | Percent |
| --- | --- | --- |
| Other (0) | 56 | 65.88 |
| Working Class (1) | 29 | 34.12 |
| N | 85 |  |

**Variable: A parent with a university degree**

|  | Freq. | Percent |
| --- | --- | --- |
| None (0) | 31 | 36.47 |
| One or more parents with a university degree (1) | 54 | 63.53 |
| N | 85 |  |

**Variable: Previous laboratory experience**

|  | Freq. | Percent |
| --- | --- | --- |
| No (0) | 72 | 84.71 |
| Yes (1) | 13 | 15.29 |
| N | 85 |  |

**Variable: Previous VR experience**

|  | Freq. | Percent |
| --- | --- | --- |
| Never (1) | 58 | 68.24 |
| Rarely (2) | 25 | 29.42 |
| Occasionally (3) | 2 | 2.35 |
| A Great Deal (4) | 0 | 0 |
| N | 85 |  |
| Mean = 1.34 |  |  |
| S.D. = 0.52 |  |  |

**Variable: Previous video gaming experience**

|  | Freq. | Percent |
| --- | --- | --- |
| Never (1) | 13 | 15.29 |
| Rarely (2) | 32 | 37.65 |
| Occasionally (3) | 27 | 31.76 |
| A Great Deal (4) | 13 | 15.29 |
| N | 85 |  |
| Mean = 2.47 |  |  |
| S.D. = 0.93 |  |  |
